# Distribution of dietary nitrate and its metabolites in rat tissues after ^15^N-labeled nitrate administration

**DOI:** 10.1038/s41598-023-28190-2

**Published:** 2023-03-01

**Authors:** Ji Won Park, Barbora Piknova, Peter J. Walter, Hongyi Cai, Supranee Upanan, Samantha M. Thomas, Khalid J. Tunau-Spencer, Alan N. Schechter

**Affiliations:** 1 Molecular Medicine Branch, National Institute of Diabetes and Digestive and Kidney Diseases, National Institutes of Health, Bethesda, MD USA; 2 Clinical Mass Spectrometry Core, National Institute of Diabetes and Digestive and Kidney Diseases, National Institutes of Health, Bethesda, MD USA

**Keywords:** Biochemistry, Physiology

## Abstract

The reduction pathway of nitrate (NO_3_^−^) and nitrite (NO_2_^−^) to nitric oxide (NO) contributes to regulating many physiological processes. To examine the rate and extent of dietary nitrate absorption and its reduction to nitrite, we supplemented rat diets with Na^15^NO_3_-containing water (1 g/L) and collected plasma, urine and several tissue samples. We found that plasma and urine showed 8.8- and 11.7-fold increases respectively in total nitrate concentrations in 1-day supplementation group compared to control. In tissue samples—gluteus, liver and eyes—we found 1.7-, 2.4- and 4.2-fold increases respectively in 1-day supplementation group. These increases remained similar in 3-day supplementation group. LC–MS/MS analysis showed that the augmented nitrate concentrations were primarily from the exogenously provided ^15^N-nitrate. Overall nitrite concentrations and percent of ^15^N-nitrite were also greatly increased in all samples after nitrate supplementation; eye homogenates showed larger increases compared to gluteus and liver. Moreover, genes related to nitrate transport and reduction (Sialin, CLC and XOR) were upregulated after nitrate supplementation for 3 days in muscle (Sialin 2.3-, CLC1 1.3-, CLC3 2.1-, XOR 2.4-fold) and eye (XOR 1.7-fold) homogenates. These results demonstrate that dietary nitrate is quickly absorbed into circulation and tissues, and it can be reduced to nitrite in tissues (and likely to NO) suggesting that nitrate-enriched diets can be an efficient intervention to enhance nitrite and NO bioavailability.

## Introduction

The roles of nitric oxide (NO) in various physiological processes have been studied since the 1980s^1,2^ and the three classes of nitric oxide synthase (NOS) enzymes were considered the main source of NO generation until nitrite (NO_2_^−^) was identified as a precursor of NO following nitrate (NO_3_^−^) ingestion in the mid-90s^3,4^. This finding began to change the older view that both nitrite and nitrate ions were largely inert end products of NO oxidation and opened a new field of research on the nitrate-nitrite-NO reduction pathway as an alternative to the classic NOS pathways. More recently it has been shown that nitrate absorbed from dietary sources such as beetroot and green leafy vegetables can be reduced to nitrite by commensal bacteria in the oral cavity^5^ and to some extent by mammalian enzymes (i.e., xanthine oxidoreductase, XOR)^6,7^. Thereafter, nitrite can be further reduced to NO by several mechanisms utilizing deoxyhemoglobin^8^, deoxymyoglobin^9,10^, several molybdenum containing enzymes^11^ and non-enzymatic mechanism^12^.

Numerous pre-clinical and clinical studies have shown that dietary nitrate supplementation can have beneficial effects in maintaining cardiovascular health and improving exercise performance, presumably by enhancing NO bioavailability^13,14^. Previous reports from rodent studies showed that providing animals with nitrite or nitrate in their diets significantly changed the endogenous concentrations of these ions in blood and organs such as heart, liver and skeletal muscle and modulated platelet function^15–18^. We also demonstrated in healthy people that increased nitrate ingestion via beetroot juice significantly elevated nitrate and nitrite concentrations in blood and skeletal muscle^19^. The transport of nitrate is known to be mediated in certain tissues by a specific transporter, sialin^20^ and also by chloride channels (CLC)^21,22^. Thus, the increase of nitrate and nitrite ions in internal organs upon nitrate ingestion may be affected by these anion transporters as well as various reductive pathways. However, dynamics of NO metabolism in mammals can be affected by the endogenous NOS pathways as well as the nitrate-nitrite-NO reduction pathways since oxidation of NOS-derived NO (and nitrite) can contribute to the systemic changes of nitrite and nitrate concentrations^23^.

We and others have examined the influences of diet and NOS on alterations of nitrate and nitrite concentrations in blood and tissues of animals by using low nitrate/nitrite diets, NOS inhibitors or a NOS-deficient mouse line^24–27^. Although these studies clearly demonstrated that both NOS and the nitrate-nitrite-NO reduction pathways modulate the concentrations of nitrate and nitrite ions in the circulation and internal organs, the information on the extent of contribution of each pathway to NO generation has not been precisely examined. To distinguish NO molecules that are produced after nitrite ingestion from those produced endogenously, Hughan et al. used ^15^N isotope and reported that the oral administration of Na^15^NO_2_ increased Hb^15^NO in healthy people^28^. In other ^15^N tracer studies, ^15^N-labeled nitrate, nitrite or arginine was administered and the levels of ^15^N species in plasma or urine samples were followed^29,30^ or whole zebrafish was used to determine the enrichment of ^15^N-labeled nitrate and nitrite^31^. However, the detailed information on the concentrations of ^15^NO_3_^−^ or ^15^NO_2_^−^ in various organs after ^15^N nitrate ingestion has not been available until now.

In the present study, we explored the dynamic changes of nitrate and nitrite concentrations in plasma, skeletal muscle, liver and eye of rats supplemented with Na^15^NO_3_-enriched water at baseline and at 1 or 3 days. Our rationale for the choice of these particular organs is that skeletal muscle (gluteus), is a nitrate storage organ^32^ and one of the beneficial effects of dietary nitrate is to enhance exercise performance^33^, while liver has relatively lower concentrations of nitrate but is abundant in Mo-containing enzymes such as XOR that are directly involved in nitrate reduction into nitrite and NO. In more recent report, we also showed that porcine ocular tissues contain considerable amount of nitrate and nitrite ions and exhibit nitrate reduction activities^34^, so eye was of our interest when examining nitrate metabolism. To evaluate the contribution of dietary nitrate on the concentrations of endogenous nitrate and nitrite, we measured the percent of ^15^NO_3_^−^ and ^15^NO_2_^−^ by LC–MS/MS in various tissue samples and also analyzed the absolute concentrations of nitrate and nitrite ions using a chemiluminescence method.

## Materials and methods

### Animals and nitrate supplementation

All animal procedures were approved by NIDDK Animal Care and Use Committee (protocol number K049-MMB-20) and carried out according to recommendations in the Guide for the Care and Use of Laboratory Animals of NIH. This animal study is reported in accordance with ARRIVE guidelines. The Wistar rats (250 ± 50 g, both male and female) were supplied by Envigo (Indianapolis, IN) and housed in a 12 h light/dark cycle environment with access to food (standard rat diet NIH07) and tap water ad libitum for 7 days before subjected to experiments. Rats were randomly divided into 3 groups and the investigators could not be blinded to the rat groups due to supplement preparation steps. Sample size of rat groups were determined based on previous reports^35,36^. The control group received tap water and the treatment groups received tap water containing Na^15^NO_3_ (1 g/L, ≥ 98 atom% ^15^N, Sigma, St. Louis, MO) for either 1 or 3 days. We analyzed the nitrite content in the Na^15^NO_3_ bottle using a chemiluminescence method and it was less than 0.01%.

### Sample collections

Rats were enclosed in an anesthesia box and deeply anesthetized using 5% isoflurane mixture with air. Anesthetized animals were placed on the pad in supine position and anesthesia continued through a nose cone. The thoracic cavity was opened and ~ 9–10 ml of blood was collected by cardiac puncture, representing about two-thirds of total blood volume for a rat of this size. Heparinized syringes were used for blood collection. Immediately after withdrawal, blood was centrifuged (17,000 g, 5 min) to obtain plasma. Animals were perfused using heparin-containing saline (Heparin Sodium, 2 USP units/ml, Hospira, Lake Forest, IL) to flush the remaining blood out of tissues. Perfusion continued until no blood was detected in outgoing saline and liver was significantly discolored. Samples from liver, gluteus muscles and eyes were then collected, placed into microcentrifuge tubes and immediately frozen on dry ice. All samples were stored at − 80 °C until analysis.

### Determination of total nitrite and nitrate concentrations

Nitrite and nitrate levels in all samples were measured using a standard chemiluminescence method as described previously^37^. Plasma and urine samples were mixed with cold methanol 1:2 (sample:methanol, vol/vol) for deproteinization, then centrifuged for 30 min (17,000 g, 4 °C, AccuSpinMicroR, Fisher Scientific, Pittsburgh, PA). The supernatant was collected and injected into the nitric oxide analyzer (NOA, Sievers, Model 280i NO analyzer, Boulder, CO) using helium as the carrier gas. Vanadium chloride or tri-iodide solution was used for nitrate or nitrite analysis respectively. All other tissue samples were weighed, mixed with water (dilution 1:5 sample:water, vol/vol) and homogenized using GentleMacs (Miltenyi Biotec Inc, Auburn, CA). Samples were centrifuged at 17,000 g for 30 min and the supernatant was collected. At this point, part of the supernatant was processed for LC–MS/MS analysis (see below) and the rest was used for NOA. NOA samples were deproteinized by dilution with cold methanol 1:2 (sample:methanol, vol/vol), then centrifuged for 30 min (17,000 g, 4 °C) to obtain clear supernatant.

### Preparation of samples for LC–MS/MS

To measure nitrate content by LC–MS/MS, nitrate ions in all samples were first reduced to nitrite enzymatically by bacterial nitrate reductase from Aspergillus niger (N7265, Sigma-Aldrich, St. Louis, MO, USA) as previously described^38,39^ with some modification. Briefly, sample (20 µl) was mixed with nitrate reductase (0.1U/ml) and NADPH (100 µM) and incubated for 2 h at room temperature. Then nitrite ions in samples were derivatized with 2,3-diaminonaphthalene (DAN, D2757, Sigma-Aldrich) for 30 min at 37 °C to yield 2,3-naphthotriazole (NAT). NaOH (58 mM) was added to terminate the reaction. For measuring nitrite content only, samples (50 µl) were directly subjected to DAN derivatization.

### Determination of ^15^NO_3_^−^ or ^15^NO_2_^−^ percent by LC–MS/MS

High-performance liquid chromatography (HPLC) grade solvents and LC–MS modifiers were purchased from Sigma-Aldrich (St. Louis, MO, USA). Detection and quantification were achieved by ultra-performance liquid chromatography—tandem mass spectrometry (UPLC-MS/MS) utilizing a Thermo Scientific Vanquish UPLC with a Thermo Scientific Altis triple quadrupole mass spectrometer, heated electrospray ionization (HESI-II) in positive ion mode (3500 V). 50 µl of sample was mixed with 200 µl of acetonitrile (ACN), vortexed for 5 min and then centrifuged at 4 °C, 17,000 g for 15 min. The supernatant was transferred to an LC–MS vial for analysis. Injection volume was 1 µl. A Waters Cortecs T3 column, 2.1 × 100 mm, 1.6 µm column was maintained at 35 °C. Solvent A: H_2_O with 0.1% formic acid (FA) and Solvent B: ACN with 0.1% FA. The flow rate was 250 µl/min, the gradient was 25% B at 0 min for 0.25 min, increasing to 65% B at 5 min, further increasing to 90% B at 5.5 min, remained 90% B until 7.5 min, then decreased to 25% B at 8 min. The total running time was 10 min. Samples were analyzed in triplicates. Quantitation of ^14^NAT and ^15^NAT were based on multiple reaction monitoring (MRM) transitions *m/z*, 170.062 → 115.042 and 171.062 → 115.042, respectively. The result was based on the percentage ratio of ^15^NAT/(^14^NAT + ^15^NAT).

### Analysis of gene expression by quantitative polymerase chain reaction (qPCR)

Total RNA from rat tissues (gluteus muscle, liver and eye, 30-40 mg each) was purified by using RNeasy fibrous tissue mini kit (Qiagen, Germany), then 1 µg of total RNA was used to synthesize cDNA by High-Capacity cDNA reverse transcription kit (Applied Biosystems, MA). For qPCR measurements, 2 µl of the synthesized cDNA, which was pre-diluted with water (1:20 ratio), was mixed with PowerUp™ SYBR™ Green master mix (Applied Biosystems, MA, USA) and primers (0.2 µM). qPCR was carried out on QuantStudio™ 7 Flex real-time PCR system (Applied Biosystems, MA). The glyceraldehyde-3-phosphate dehydrogenase (*gapdh*) gene was used as a reference gene to calculate relative quantification by the 2^−ΔΔCt^ method.

The primer sequences used for qPCR were as follows: *sialin* forward (ACACTCTGCCCCCGTAAAAG) and reverse (TCTGTGTGACGATGTAGCCG), *clc1* forward (ACAATGCCCACCCAACACA) and reverse (GTCCTCATCCAAGCTGTCCA), *clc2* forward (AGATTGTCCAGGTGATGCGG) and reverse (TGAACTGTCCAAAGCCAGGG), *clc3* forward (CGTGTCCCGGTGAAGCTC) and reverse (CAGCTGCTCAGACTCCATGT), *xor* forward (TGACTGCGGATGAGTTGGTC) and reverse (AAGCTTGGTCCCACATAGCC), *gapdh* forward (CCGCATCTTCTTGTGCAGTG) and reverse (ATGAAGGGGTCGTTGATGGC).

### Statistical analysis

Values represent average ± standard deviation. Statistical significance of results was tested using the one-way ANOVA. * denotes *p* < 0.05.

## Results

**Figure 1:**
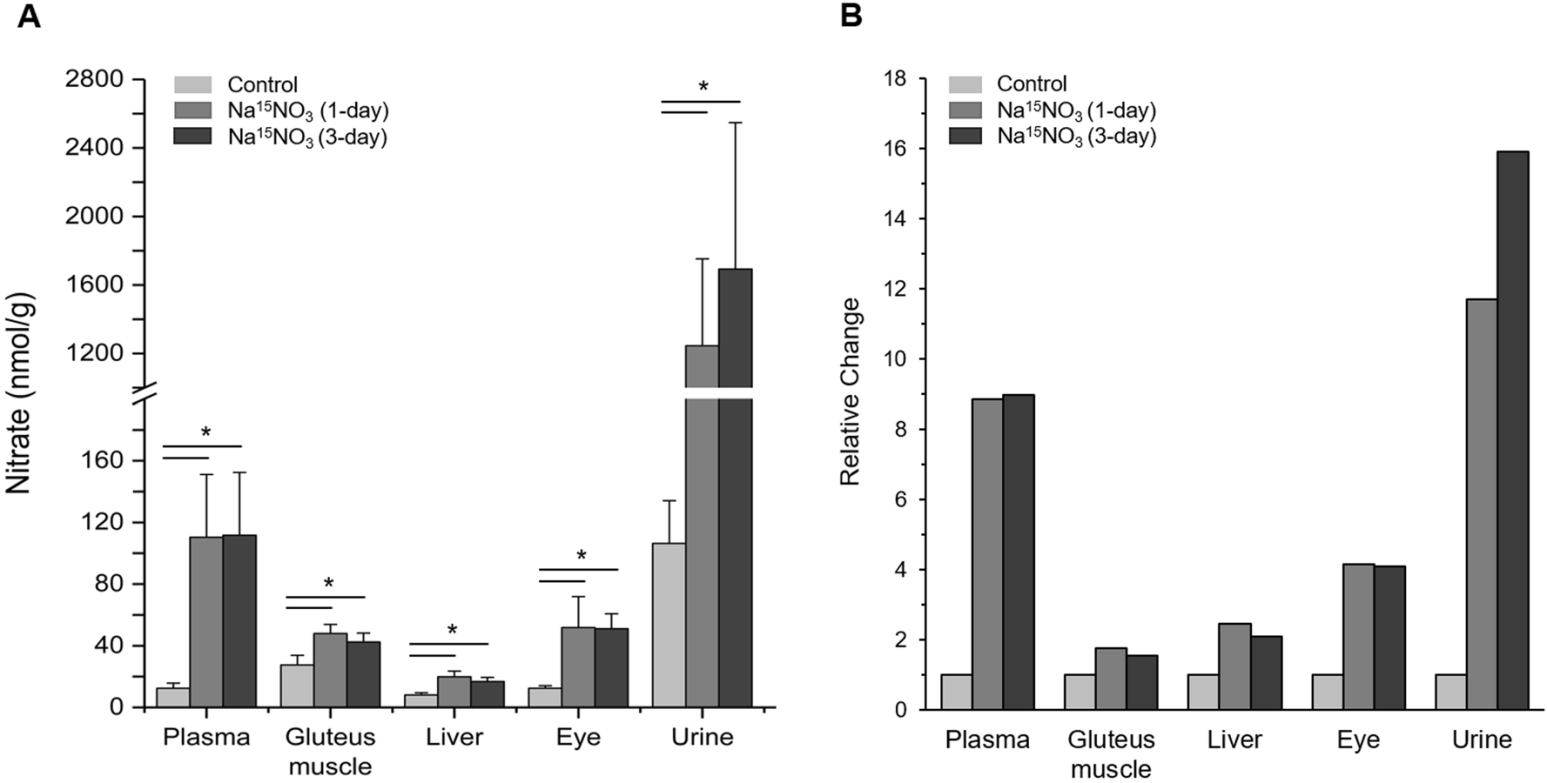
Total nitrate concentrations in rat tissues. (**A**) Tissue samples were homogenized by a rotary homogenizer, then centrifuged (17,000 g, 30 min) after mixing with methanol for deproteinization^40^. The supernatant was used for nitrate measurement by a standard chemiluminescence method with vanadium chloride. Data are plotted as average ± standard deviation (n = 4–6, * *p* < 0.05). (**B**) The change in each organ at 1-day and 3-day Na^15^NO_3_ supplementation (1 g/L) relative to control.

To assess the influence of dietary nitrate delivered via drinking water on the concentrations of nitrate and nitrite in various tissues of rats, we provided animals with ^15^N-labeled nitrate water (Na^15^NO_3_, 1 g/L) for 0, 1 or 3 days. The total amount of nitrate and nitrite in plasma, urine and tissue samples (gluteus muscle, liver and whole eye) was measured by a standard chemiluminescence method^40,41^. Nitrate concentrations increased significantly in all samples of 1-day supplementation group compared to the baseline of control group (no treatment) and remained elevated in 3-day supplementation group (Fig. 1A). The fold change of nitrate concentrations in plasma and urine was 8.8- and 11.7-fold increase respectively compared to control and other tissue samples, gluteus muscle, liver and eye homogenates, also exhibited a significantly raised nitrate concentrations in 1-day supplementation group compared to control (1.7-, 2.4- and 4.2-fold increase respectively, Fig. 1B). These changes remained similar in all these sample tissues in 3-day supplementation group.

**Figure 2:**
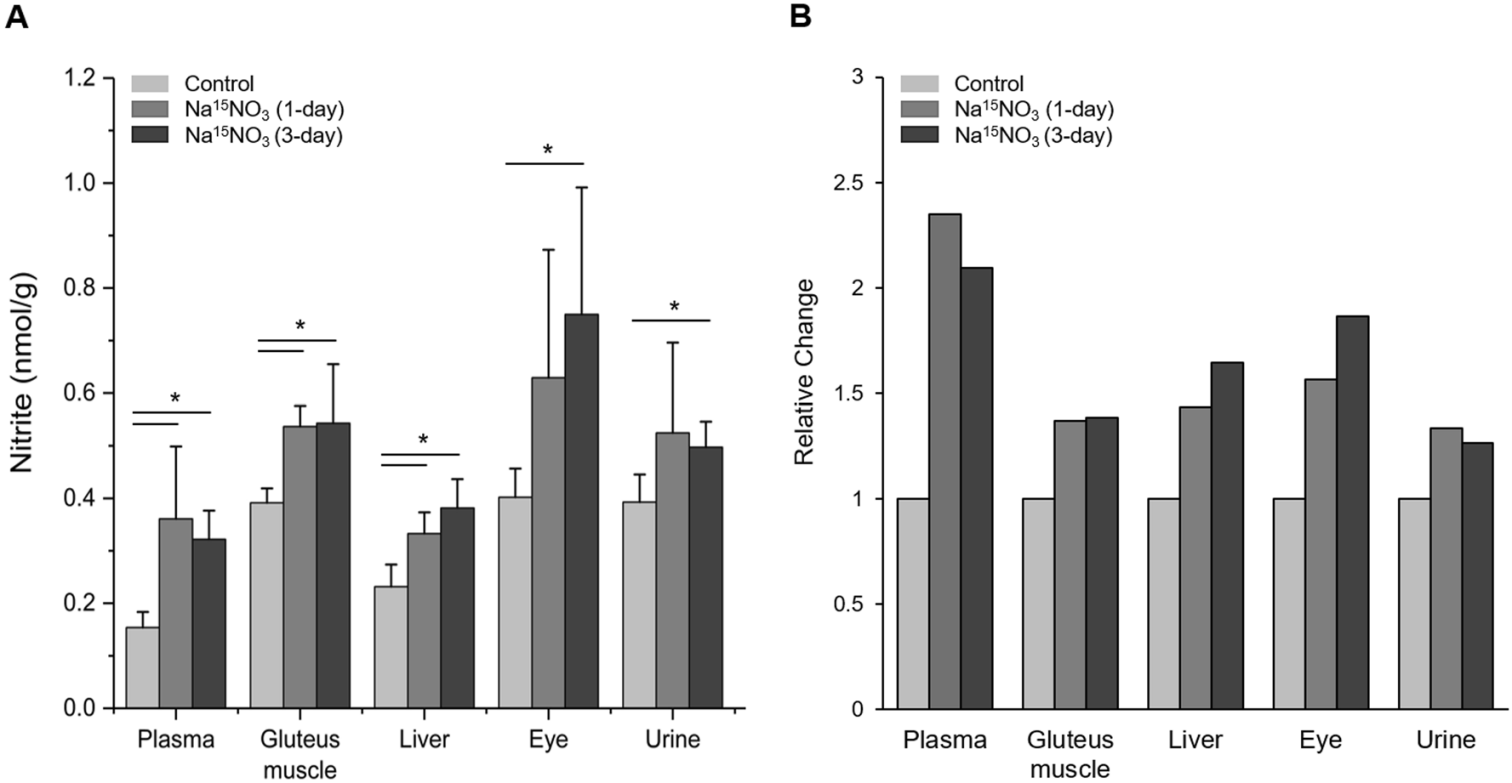
Total nitrite concentrations in rat tissues. (**A**) Tissue samples were homogenized by a rotary homogenizer^40^, then centrifuged (17,000 g, 30 min) after mixing with methanol for deproteinization. The supernatant was used for nitrite content measurement using a standard chemiluminescence method with triiodide. Data are plotted as average ± standard deviation (n = 4 ~ 6, * *p* < 0.05). (**B**) The change in each organ at 1-day and 3-day Na^15^NO_3_ supplementation (1 g/L) relative to control.

Since nitrate ions can be reduced to nitrite ions by several mechanisms once it is absorbed into blood and tissues, we measured the concentrations of nitrite in tissue samples as well as in plasma and urine (Fig. 2A). In plasma, gluteus muscle and liver samples, there was a significant rise in nitrite concentrations after 1- and 3-day supplementation, while eye and urine showed an increase after 1-day supplementation and it was statistically significant increase after 3-day supplementation. In fold change analysis, plasma showed the biggest increases (2.3- and 2.1-fold increase at 1-day and 3-day supplementation respectively) but eyes (1.6- and 1.9-fold increase), liver (1.4- and 1.6-fold increase) and gluteus muscle (1.4- and 1.4-fold increase) also exhibited notable increases in nitrite concentrations (Fig. 2B). Tissue samples suggested further small increases at 3-day compared to 1-day supplementation.

**Figure 3:**
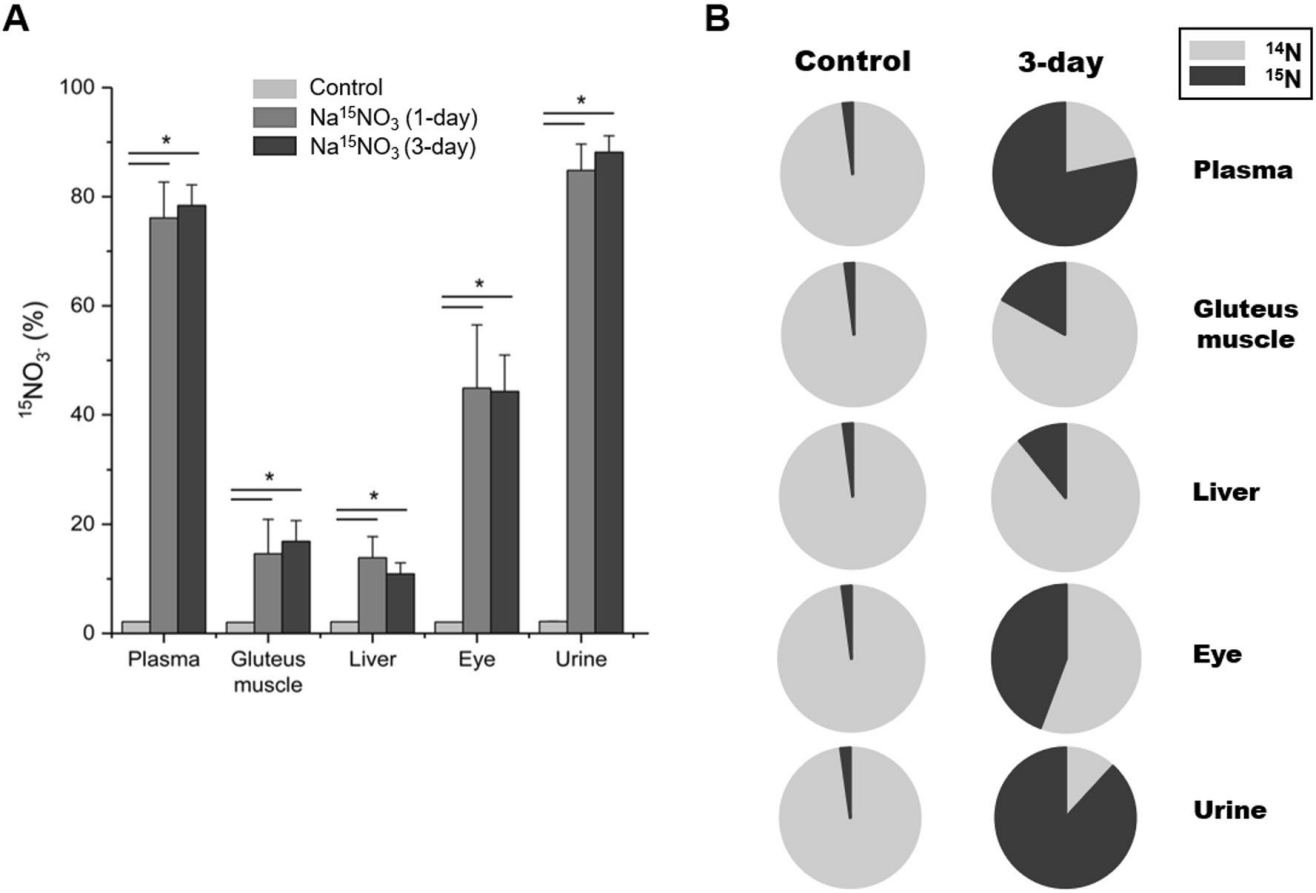
Relative enrichment of ^15^N-nitrate in tissues after dietary intervention analyzed by LC–MS/MS. (**A**) ^15^N-labeled nitrate content was measured by LC–MS/MS. All nitrate ions in samples were first reduced to nitrite by bacterial nitrate reductases (Aspergillus niger). Then nitrite derivatization by DAN was performed to yield NAT. The result was based on the percentage ratio of ^15^NAT/(^14^NAT + ^15^NAT). Data are plotted as average ± standard deviation (n = 4–6, * *p* < 0.05). (**B**) The proportion of ^15^N-labeled nitrate in control and 3-day Na^15^NO_3_ supplementation group is shown.

**Figure 4:**
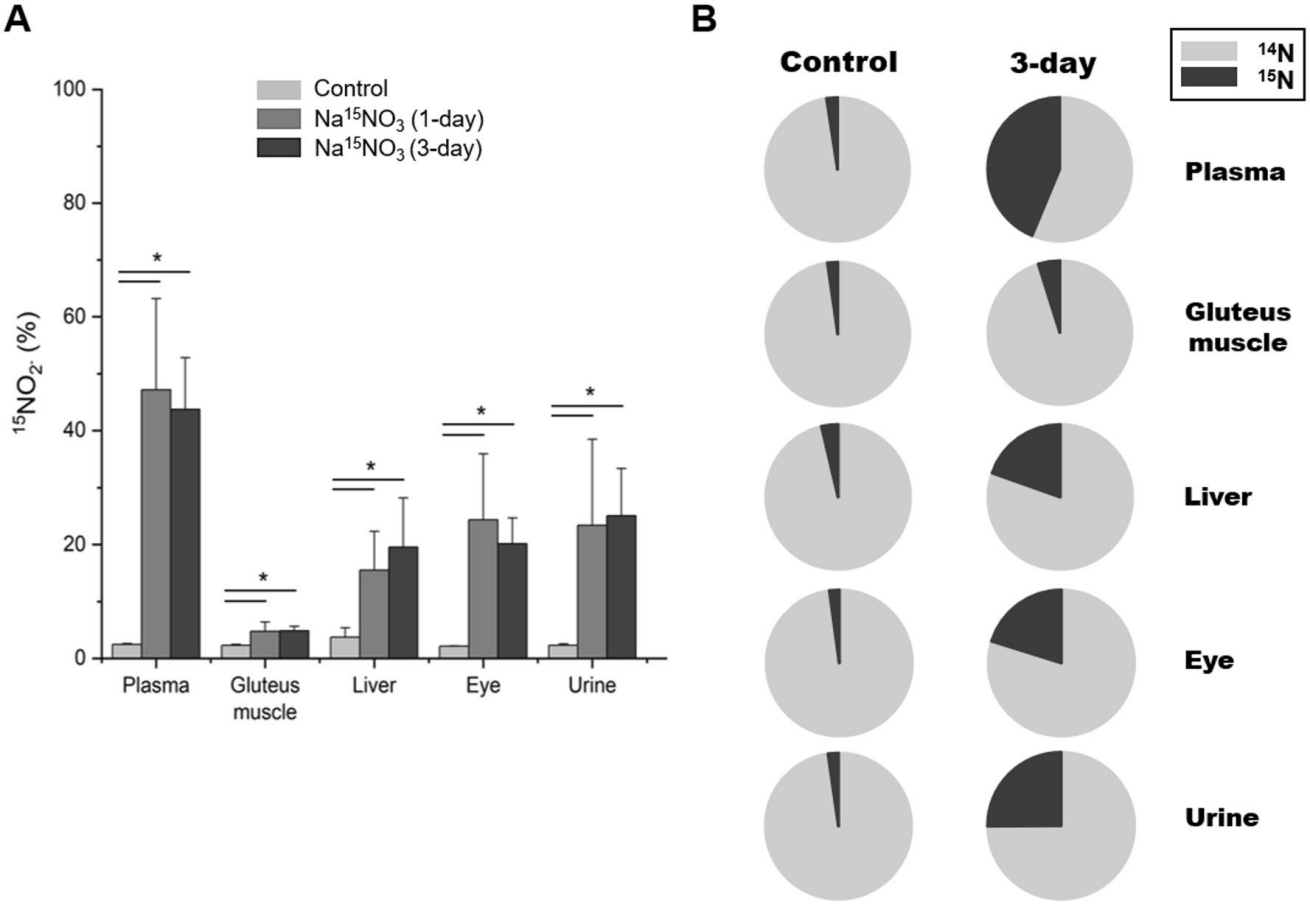
Relative enrichment of ^15^N-nitrite in tissues after dietary intervention analyzed by LC–MS/MS. (**A**) ^15^N-labeled nitrite content was measured by LC–MS/MS. Nitrite derivatization by DAN was performed to yield NAT. The result was based on the percentage ratio of ^15^NAT/(^14^NAT + ^15^NAT). Data are plotted as average ± standard deviation (n = 4–6, * *p* < 0.05). (**B**) The proportion of ^15^N-labeled nitrite in control and 3-day Na^15^NO_3_ supplementation group is shown.

Although it is reasonable to presume that the supplemented nitrate via drinking water is responsible for the elevation of endogenous nitrate and nitrite concentrations as shown in Figs. 1 and 2, it is important to confirm the direct contribution of dietary nitrate to the concentrations of nitrate and nitrite in tissues to distinguish it from the NOS-derived nitrite and nitrate formation. To precisely analyze how much nitrate and nitrite is coming from the supplemented ^15^N-labeled nitrate, we measured ^15^N-labeled nitrate and nitrite content by LC–MS/MS. Figures 3A and 4A show the percent of ^15^N nitrate and nitrite respectively in three tissue samples as well as plasma and urine. In 1-day supplementation group, 76% of total plasma nitrate was ^15^N nitrate and 85% of total urine nitrate was ^15^N nitrate and these were remained very similar in 3-day supplementation group. Gluteus muscle, liver and eye homogenates also showed a remarkable increase in ^15^N nitrate percent (15, 14 and 45% respectively in 1-day supplementation group) (Fig. 3A). The percent of ^15^N nitrite was also significantly elevated in all tissue samples after ^15^N nitrate supplementation and plasma had the highest percent of ^15^N nitrite (47 and 44% at 1-day and 3-day supplementation). Among tissue samples, eye had higher ^15^N nitrite levels (24 and 20% at 1-day and 3-day supplementation) than gluteus muscle (4.8% at both 1-day and 3-day supplementation) and liver (15 and 20% at 1-day and 3-day supplementation) (Fig. 4A). Figures 3B and 4B show the proportion of ^15^N nitrate and nitrite respectively in control and 3-day supplementation group.

**Figure 5:**
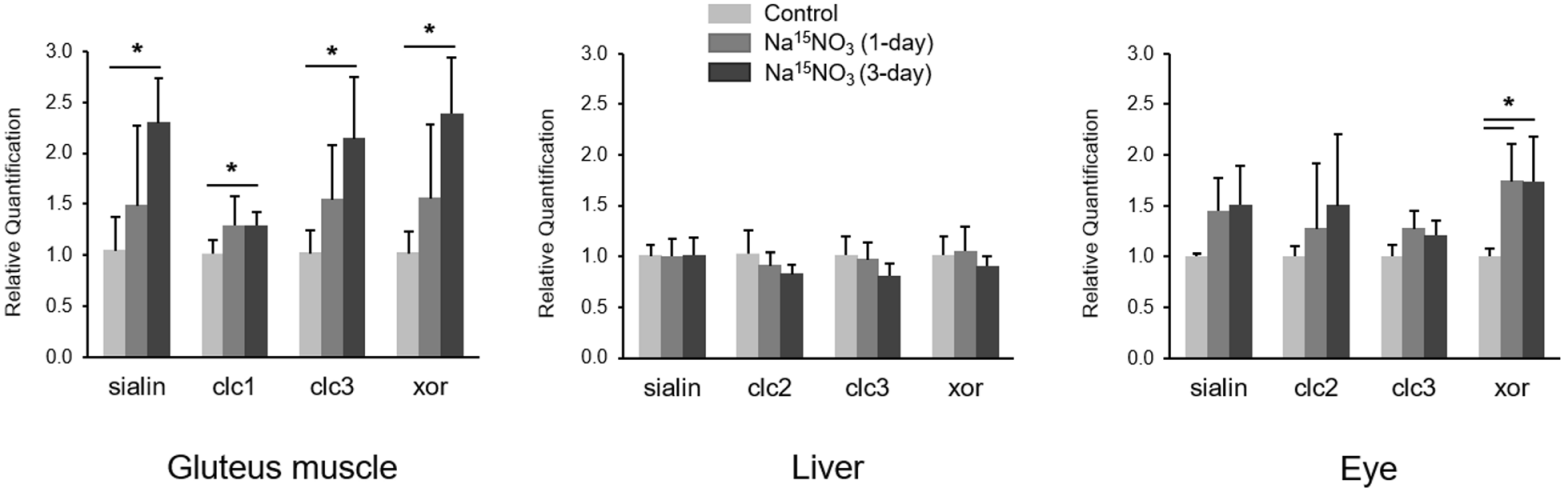
Gene expression analysis. Total RNA was isolated from tissue samples (gluteus skeletal muscle, liver and eye) of control, 1- and 3-day Na^15^NO_3_ supplementation group and used for cDNA synthesis. The levels of mRNA were determined by qPCR and relative quantification was calculated by the 2^−ΔΔCt^ method using *gapdh* as a reference gene. Data are plotted as average ± standard deviation (n = 3–6, * *p* < 0.05).

To examine the expression changes in genes related to nitrate transport and reduction pathway, we performed a qPCR assay with gluteus muscle, liver and eye homogenates (Fig. 5). We assessed the expression levels of sialin, CLC (nitrate transporters) and XOR (nitrate reductase). In gluteus muscle, there was a significant increase in sialin, CLC1 (skeletal muscle-specific isoform of CLC), CLC3 and XOR expression levels in 3-day supplementation group. We observed a trend of increases in sialin, CLC2 and CLC3 in eye homogenates and a significant rise in XOR expression levels in 1-day and 3-day supplementation group. However, in liver, there was no change in these genes after nitrate supplementation.

## Discussion

Since the beneficial effects of dietary nitrate as NO source are well documented, particularly in enhancing cardiovascular health and exercise performances^42,43^, it is evident that the contribution of diet to NO homeostasis plays a big role in various physiological processes. Importantly, the endogenous NOS requires oxygen for its activity when producing NO, but the nitrate-nitrite-NO reduction pathway can be augmented under hypoxic environment^44^, which makes it a valuable alternative to the other NO generation mechanism. To elucidate the physiological importance and direct contribution of dietary nitrate to the concentrations of nitrate and nitrite as NO source, we employed ^15^N-labelled nitrate salts in drinking water to trace both ^15^N nitrate and ^15^N nitrite in several different tissues. Although there have been a few studies where stable isotopes were used to detect ^15^N nitrate and nitrite in body fluids such as plasma and urine, more detailed information on the levels of these isotopes in major organ samples after consuming ^15^N-labelled nitrate salts is not available so far. To our knowledge, this is the first study that shows the distribution of both ^15^N nitrate and nitrite in plasma, urine and tissues from rats supplemented with ^15^N-labelled nitrate salts and provides direct evidence of the reductive pathway for NO generation mediated by dietary nitrate in mammalian tissues. It also provides a short glimpse into the interesting complexity of nitrate uptake dynamics into internal organs, with a somehow unexpected speed of dietary nitrate influx into these tissues. In principle, isotope studies will allow to follow nitrate uptake kinetics into various organs and evaluate similarities/differences between various tissues.

In our recent studies in rats, we identified skeletal muscle as a nitrate-enriched organ compared to liver or blood and showed that nitrate, stored in skeletal muscle, could be utilized to produce nitrite and NO after exercise^7,32^. Consistent with previous results, we show in the current study that skeletal muscle (in particular, gluteus) contained higher nitrate concentrations (27.5 nmol/g) than other tissues (liver and eye, 8.1 and 12.5 nmol/g respectively) and plasma (12.5 nmol/g) at baseline (Fig. 1A). However, this nitrate concentration gradient, from muscle to plasma and other organs, in the steady state did not persist after nitrate supplementation. Nitrate concentrations were much higher in plasma (105.1 nmol/g) than other tissues (47.9, 19.8 and 51.8 nmol/g in gluteus, liver and eye respectively) except urine (1245.0 nmol/g) in 1-day supplementation group and the same pattern remained in 3-day supplementation group. Likely, high concentrations of nitrate in the circulation are important to distribute these ions to specific places where needed. Urine was previously identified as an important route to excrete excess nitrate out of body. It was shown that approximately 60% of administered nitrate is excreted in the urine in 24–48 hours^45^ and we also observed very high concentrations of nitrate in urine samples both at 1-day (1245.0 nmol/g) and 3-day (1693.3 nmol/g) supplementation. It is noteworthy and somewhat surprising that eyes absorbed nitrate ions more efficiently than gluteus muscle and liver when comparing relative changes after nitrate supplementation (Fig. 1B). Interestingly, recent papers showed an inverse association of dietary nitrate intake with primary open-angle glaucoma and age-related macular degeneration suggesting that a higher nitrate consumption may lower the risk of getting such ocular diseases^46,47^. While NO signaling governed by NOS was reported to be involved in the regulation of ocular blood flow and pressure^48–50^, very little information is currently available regarding the effects of nitrate and nitrite as NO precursors in eye physiology. To elucidate potential contributions of nitrate to NO pathways in the eye, we previously demonstrated that porcine ocular tissues, particularly cornea and sclera, possess the ability to reduce nitrate to nitrite suggesting that a functional nitrate-nitrite-NO pathway exists in the eye^34^. In that study, we postulated that lacrimal glands absorb nitrate from blood and secret it into the eye where it can be reduced to nitrite and NO by several mechanisms and this function of lacrimal glands seems to resemble well-known roles of salivary glands on concentrating and secreting nitrate into the oral cavity. In the present study, we measured nitrite and nitrate concentrations in whole eye homogenates after ^15^N nitrate supplementation to extend our knowledge on nitrate metabolism in the eye. We showed that eye homogenates exhibited 1.9-fold increase in absolute nitrite concentrations while gluteus and liver showed 1.4- and 1.6-fold increase in 3-day supplementation group (Fig. 2B) and more importantly, the ^15^N nitrite content at 3-day supplementation was 20.1, 19.6 and 4.8% in eye, liver and gluteus respectively (Fig. 3B). The difference between eye and other tissues was even more notable in ^15^N nitrate content (Fig. 3A). In 3-day supplementation group, 44.3% of total nitrate was ^15^N nitrate in the eye, while 16.9 and 10.9% was ^15^N nitrate in gluteus and liver. This suggests that eyes are more sensitive to taking dietary nitrate ions up and have ability to metabolize it. However, more detailed study is required to understand the precise mechanism by which nitrate ions are utilized and play a role in eye physiology and perhaps the pathophysiology of some ocular diseases.

The ^15^N nitrite detected in tissue samples could be derived from two sources, namely the reduction from supplemented ^15^N nitrate in situ or transport from the circulation which was already reduced from nitrate in the oral cavity or in other tissues. Although we could not estimate the extent of contribution by each source in the tissue samples, these results demonstrate that the substantial amount of dietary nitrate can be reduced to nitrite in the body and be utilized in specific tissues or re-distributed according to the needs.

We previously showed that the concentrations of several proteins related to nitrate metabolism, such as XOR and sialin, changed in rat skeletal muscle upon perturbation of nitrate supply^18^. Additionally, we and others show that knock-out mice, specifically myoglobin and eNOS knock-out mice, have higher expression levels and activities of XOR^51,52^. Although oral commensal bacteria are thought to be the main contributor to nitrate reduction to nitrite upon dietary nitrate consumption, several molybdenum-containing enzymes were suggested to be able to reduce nitrate, particularly XOR was shown to function as a nitrate reductase in mammalian tissues^6^. We also demonstrated that the inhibition of XOR almost completely prevented nitrate reduction to nitrite in rat skeletal muscle homogenates^7^. However, it is also important to note that there are significant differences between human and rodents in nitrate reduction capacity by commensal bacteria^53,54^. This suggests that mammalian enzymes such as XOR might be more important in reducing nitrate and nitrite in rodents than it is in humans, at least in some tissues. To examine whether XOR expression levels were altered by nitrate administration, we performed a qPCR analysis and observed a significant increase of XOR gene in gluteus muscle and eye in 3-day supplementation group (Fig. 4). Interestingly, we noted an increase in expression levels of nitrate transporter, sialin and CLC as well in gluteus and eye, although the difference was not statistically significant in the eye (Fig. 4). These results suggest that there is a positive feedback loop between nitrate and its transport system which can eventually augment the nitrate-nitrite-NO pathway in these tissues.

**Figure 6:**
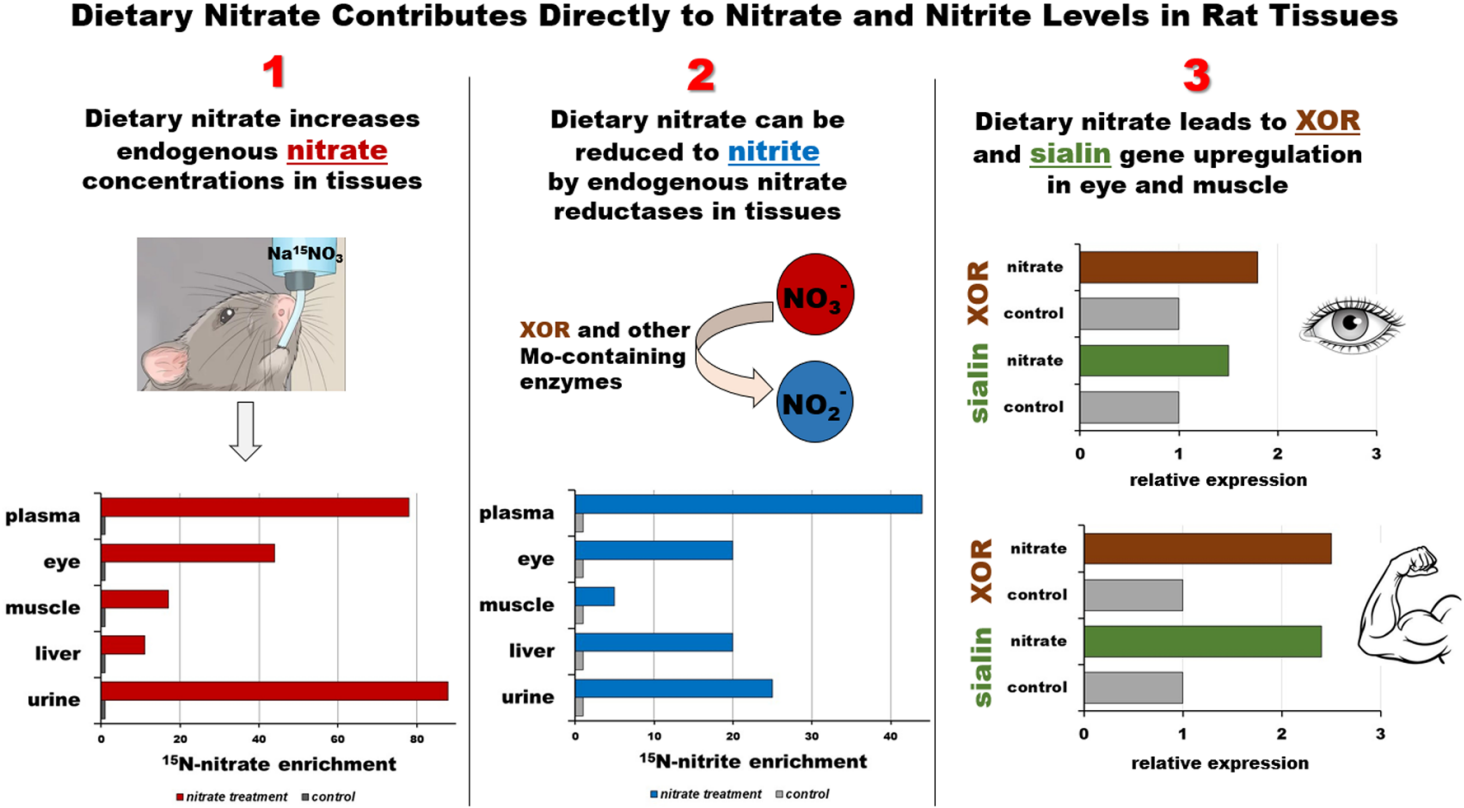
Effects of dietary nitrate supplementation on distribution of nitrate and nitrite ions and gene expression levels in tissues. 1: Na^15^NO_3_ (1 g/L) supplementation for 3 days via drinking water lead to significant increases of ^15^N nitrate contents in rat tissues and plasma. Unabsorbed ^15^N nitrate is excreted in the urine. 2: Endogenous nitrate reductases, such as XOR, can reduce ^15^N nitrate to ^15^N nitrite in tissues and cause a rise in ^15^N nitrite concentrations. Other Mo-containing enzymes include aldehyde oxidase (AO), sulfite oxidase (SO) and mitochondrial amidoxime reducing component (mARC). 3: Na^15^NO_3_ (1 g/L) supplementation for 3 days induced an upregulation of nitrate transporter (Sialin) and nitrate reductase (XOR) genes in eye and gluteus muscle.

In summary, we analyzed both absolute concentrations and ^15^N percent of nitrate and nitrite ions after Na^15^NO_3_ administration in drinking water and showed that nitrate supplementation led to avid uptake of nitrate in rodent tissues, such as muscle, liver and eye and the conversions of nitrate to nitrite occur in these tissues. Our study is the first to estimate the rate and extent of absorbed exogenous nitrate and its reduction to nitrite in major rodent organs by using ^15^N-labeled nitrate salts and LC–MS/MS analysis. A summary in Fig. 6 shows the effect of nitrate supplementation on distribution of nitrate and nitrite ions and gene expression in tissues as discussed in this study. These results demonstrate that supplying nitrate via drinking water is an efficient way of increasing nitrate and nitrite concentrations in tissues and is likely to contribute to improving NO bioavailability in these tissues.

## Data Availability

The dataset used and/or analyzed during the current study is available from the corresponding author on reasonable request.
